# The use of animals in physiological science: the past, the presence, and the future

**DOI:** 10.1007/s00424-024-03009-9

**Published:** 2024-08-28

**Authors:** Klaus-Dieter Schlüter

**Affiliations:** Physiologisches Institut, Justus-Liebig-University Giessen, Aulweg 129, D-35392 Giessen, Germany

**Keywords:** 3R, Animal testing, Ethics

## Abstract

Physiology is a scientific discipline of how people’s and animals’ bodies function that requires traditionally suitable experimental models that often rely on animals. However, at the end of the 50th of the last century, researchers themselves addressed concerns about the use of animals for biomedical science and physiology in particular. At that time, the so-called 3R strategy was implicated where the three “R” stand for replacement, reduction, and refinement. When addressing these concerns, researchers nevertheless realized that a critical dispute about experimental models in the light of the 3R initiative may require further attention to other points such as robustness, registration, reporting, reproducibility, and rigor of the work. The question that has to be addressed now is first whether the use of animals in physiology changed in the post-3R period, whether it led to a replacement, reduction, or refinement of animal handling, and most importantly, how this affected the scientific progress in (patho)physiology. In order to address open questions concerning the relationship between the use of animals and physiological research, complete volumes of the *Pflügers Archiv – European Journal of Physiology* were analyzed every 10 years starting in 1950 and ending in 2020 and compared to volumes of the *Journal of Physiology*. It analyzed how scientists organize their projects published in the journal and what kind of models they used. The results show that physiological science has dramatically changed in the last 70 years. Replacement, reduction, and refinement were achieved to a certain level. However, during the last years, no further achievement could be seen. It seems that a certain level of animal testing is required for biomedical science and physiology in particular. Physiological studies in the present time are dominated by investigation of the physiological function of small rodents mainly mice and rats with only a few exceptions. The analysis also shows that in the future, researchers must have a critical look at further requirements of their research such as data robustness, improvement of reproducibility of data, and generation of rigor data as a prerequisite to improve our physiological view on life.

## Introduction

Physiology is a scientific discipline of how people’s and animals’ bodies function, and of how plants function. Results depicted from physiological studies using human volunteers and/or animals generate new treatment strategies that are aimed at curing chronic diseases. To achieve these aims, it is important to identify the underlying mechanisms of diseases. This part of physiological science is called pathophysiology. However, in practice, the disciplines of physiology and pathophysiology overlap. Without physiological knowledge, no pathophysiological concepts can be established. Both physiological and pathophysiological research require proper experimental models that mostly depend on animals. Historically, physiologists worked either with volunteers or highly developed mammalians such as dogs, cats, and horses [3, 4]. Insight and outside the research family, however, people set question marks about animal testing. This discussion started approximately 100 years ago and is still ongoing [7]. At the end of the 50th of the last century, researchers themselves addressed concerns about the use of animals for biomedical science and in physiology in particular [5]. At that time, the so-called 3R strategy was implicated where the three “R” stand for replacement, reduction, and refinement [10]. Criteria were announced aimed at reducing the use of animal testing either completely and replacing it with other methods such as computer modeling (replacement strategy) or aimed at reducing stress to the animals (reduction) or minimizing animal suffering enhancing the welfare of animals in research situations (refinement). Although initiated in the early second half of the last century, 3R criteria did neither play a visible role in scientific education nor in physiological research for many years but with the increasing criticism of animal testing they got more and more attention in the last years. As a matter of fact, they influenced the EU directive for animal use in science and subsequently the registration processes in most countries with active physiology research centers [6]. However, it is not clear how the introduction of the 3R criteria changed physiological research. Successful physiological research projects require three different steps. First, they require a specific scientific question that should be addressed; second, they need a hypothesis on how the question may be addressed experimentally; and finally, they require a selection of a valid and useful experimental model. If these three steps are fulfilled, successful research projects lead to the publication of the data in a peer-reviewed journal. Acceptance of a peer-reviewed manuscript means that the scientific community has accepted the study as important and complete. Such a study significantly improves our present knowledge in biomedicine. Keeping this in mind, researchers recognized that the 3R criteria are not sufficient to judge about the usefulness of animal testing. Indeed there are more points that need to be addressed such as robustness, registration, and reporting (called 3R + 3R), as well as reproducibility and rigor (called 3R + 2R) of the research [2, 9, 12]. Furthermore, in 2020, further suggestions as outlined in ARRIVE 2.0 aimed at improving at least the reproducibility of animal testing [8]. In the last few years, researchers started to investigate how these things affect the quality of their work and the progress of biomedical research. However, data addressing the question of how animal testing has changed in the last years in physiology have not been analyzed so far. The principle questions are still unanswered: Did the use of animals in physiology change in the post-3R period, did this lead to a replacement, reduction, or refinement of animal handling, and how does this affect the scientific progress in (patho)physiology?

The *Pflügers Archiv – European Journal of Physiology* is an international recognized journal focusing on physiologic research. The journal was founded in 1868 by Eduard Pflüger and published studies today under the supervision of the German Society of Physiology. It reports about the complete physiology of humans and animals. It is currently ranked among the Q1 journals of this scientific discipline. The long tradition of this journal in connection to its still high visibility makes it possible to investigate changes in physiological research and to analyze how animals are used in this biomedical discipline. In order to address open questions concerning the relationship between the use of animals and physiological research, complete volumes of the *Pflügers Archive – European Journal of Physiology* were analyzed in this study every 10 years starting in 1950 and ending in 2020. It was analyzed how the authors organized their projects published in the journal and what kind of models they used. The data show how physiology has incorporated the ideas of 3R and improved our research in the past, and this gives us a basis to discuss how physiology as a scientific discipline will further develop in the future. To investigate whether the data obtained by an analysis of the *Pflügers Archiv – European Journal of Physiology* are representative of physiology, the data were also compared to the *Journal of Physiology*, a journal founded in 1878 by Michael Foster and published in the name of the *Physiological Society*.

## Material and methods

Complete volumes of the *Pflügers Archiv – European Journal of Physiology* were analyzed covering the years 1950, 1960, 1970, 1980, 1990, 2000, 2010, and 2020. Only research articles in regular issues (excluding research articles in focused issues to exclude technical and thematic bias) were analyzed. Questions under investigation were first whether the authors performed animal experiments (called in vivo), whether they performed experiments with tissues, cells, or organs from animals (called ex vivo), whether they performed experiments with material not specifically isolated from animals or humans for their specific studies (i.e., use of cell lines called in vitro), whether they used healthy volunteers (called patient based studies), or whether they performed studies without biological material (called computer science or development of new techniques). In cases in which different methods were used in one study, the study counts for the technique with the highest stress level to animals, i.e., if in vivo experiments were reported together with ex vivo and in vitro experiments, the study is claimed as in vivo study, as the main conclusion depended on animal experiments. The second part of the analysis deals with the question of what kind of species the researchers used for these studies, followed by questions regarding the number of animals used for the studies and the questions whether the animals were sacrificed at the end of the study or not. A third part investigated whether an ethics approval was communicated or whether it was at least mentioned that the study was performed in accordance with generally claimed procedures. Furthermore, it was analyzed how researchers organized their work, i.e., how many researchers cooperated and whether this was done in a multi-center approach or based on individual institutes.

Finally, it was investigated whether the results obtained by the analysis of manuscripts published in the *Pflügers Archiv – European Journal of Physiology* can be compared to other international journals focusing on physiology. Here, the data were compared to those from the *Journal of Physiology*, a journal founded in 1878 and also publishing data from all areas of physiology. Again, complete volumes of the Journal were analyzed from 1960, 1980, 2000, and 2020 and compared with those obtained above.

## Results

### Publication strategies and authors

**Fig. 1:**
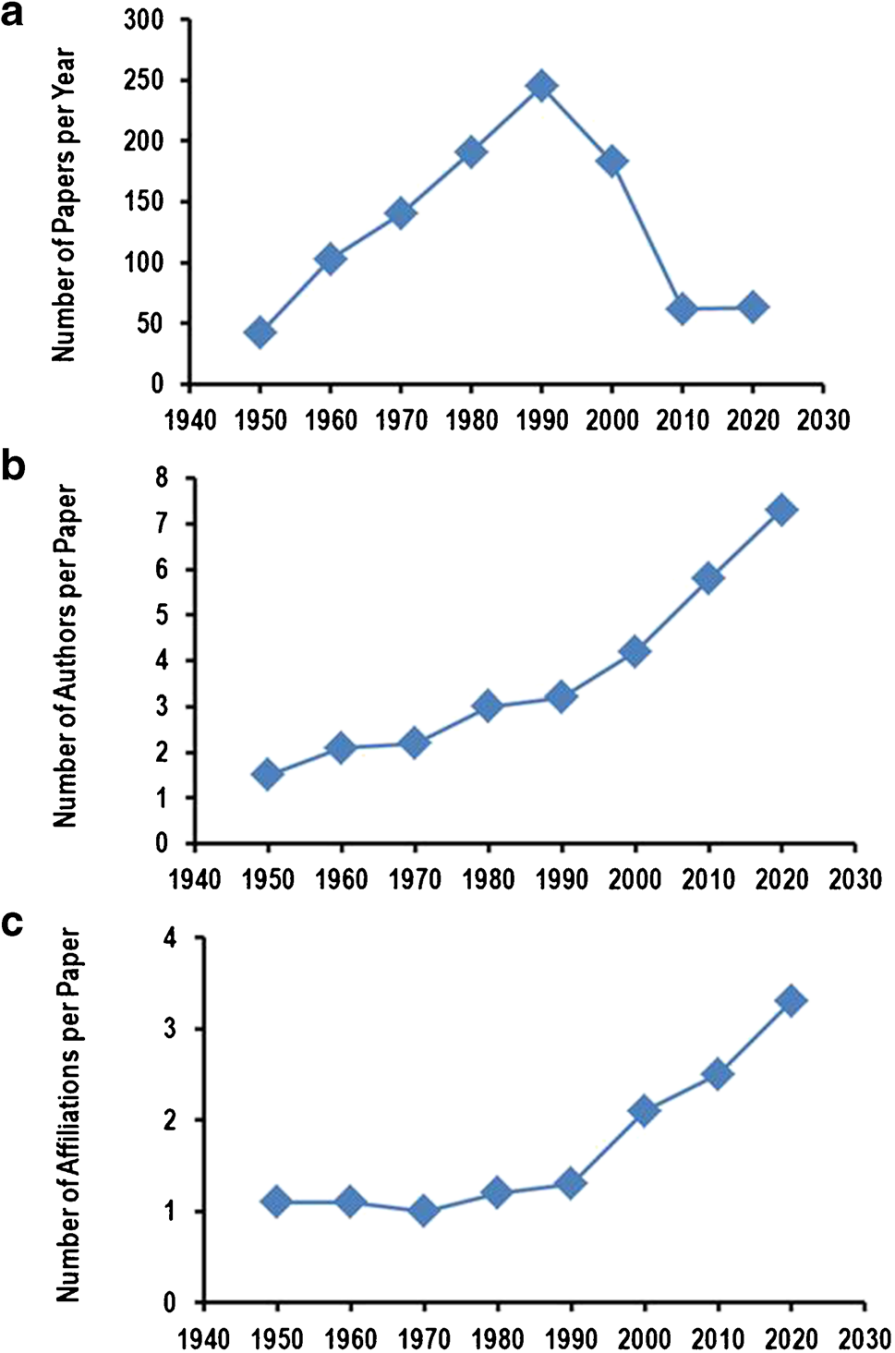
Publication strategies and authors in research papers of *Pflügers Archiv – European Journal of Physiology*. **A** Number of research papers published per year. **B** Number of authors per publication. **C** Number of affiliations for authors per publication

Starting from 1950, the number of papers published in the journal increased from 42 in 1950 up to 245 original research papers published in 1990 (Fig. 1A). From that time on, the number of published studies was significantly reduced to approximately 62 per year in our days. However, new types of manuscripts entered the journal at the same time such as review papers, editorials, and viewpoints that discuss physiological findings in a brought way.

A remarkable increase in the number of authors that participated in individual studies can be observed. Whereas 1.5 authors per report published data leading to individual research papers in 1950, the number increased to 7.3 authors per report in 2020 (Fig. 1B). This means that in 2020, 460 authors reported data in only 63 research papers, whereas in 1990, only 319 authors reported their data but produced 245 papers. In other words, although the number of publications is lower in our days, more researchers participate in publications of the journal. Moreover, authors from different laboratories cooperate in our days to generate data as indicated by an increase in the number of affiliations of authors per paper (Fig. 1C).

### In vivo and in vitro studies

**Fig. 2:**
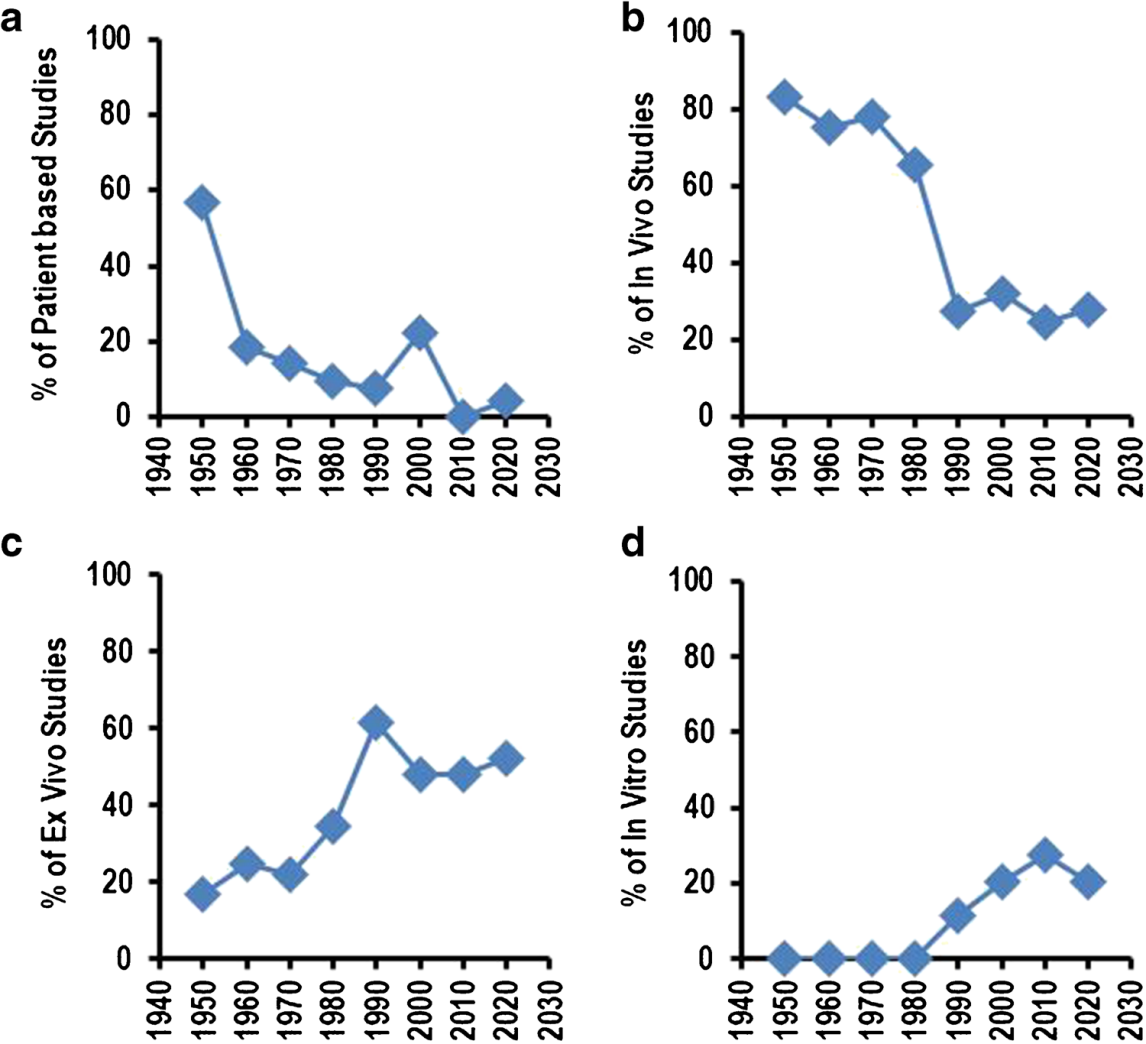
Type of studies in research paper published in *Pflügers Archiv – European Journal of Physiology* per year. **A** Patients or volunteer-based studies expressed as % of all research papers. **B** In vivo studies (classical animal studies) expressed as % of all animal-based studies. **C** Ex vivo studies (isolated organs) expressed as % of all animal-based studies. **D** In vitro studies (cell culture-based studies) expressed as % of all animal-based studies

Traditionally, physiological research is based on animal studies or patient-based studies. This is clearly visible in the high proportion of volunteer-based studies in 1950 and the high number of in vivo studies (Fig. 2A, B). There is only a small amount of ex vivo studies in 1950 (16.7%), and no in vitro studies were published at that time (Fig. 2C, D). The proportion of ex vivo studies increased from 1970, reaching a stable proportion from 1990 to 2020 of approximately 50% (Fig. 2C). Starting from 1990, in vitro studies were accepted in physiological research, and they represent today approximately 20–25% of all studies (Fig. 2D). In contrast, patient-based physiological research has dramatically reduced from 1960 on. In vivo studies with animals accounted initially for more than 80% of all animal-based studies, but this proportion declined to 20–25% until 1990 with no further decrease until 2020 (Fig. 2B). Computer-based analysis and/or studies reporting new techniques such as the development of electrodes do not play a significant role at present, although in rare cases, such studies were published. In summary, ex vivo and in vitro studies largely replaced classical patient-based studies or in vivo animal-based studies.

### Use of species in physiological science

**Fig. 3:**
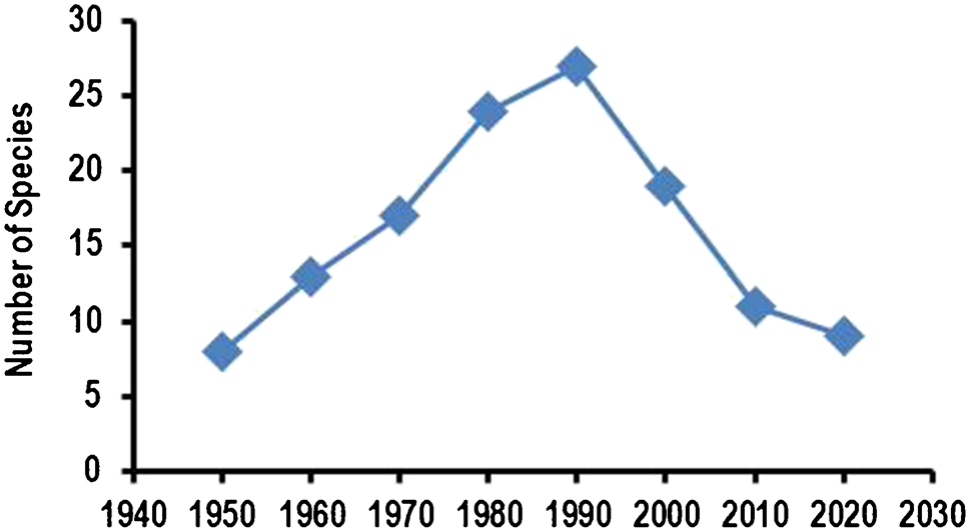
Number of animal species used in animal-based studies per year

In general, all types of animals from insects, nematodes, and vertebrates to primates are feasible models for physiological research. However, depending on the scientific question, they have probably different usability for a given project. For example, evolutionary old principles work in low-level developed animals such as insects as well as in highly developed animals like primates. Therefore, there will be no need to use primates to address such questions. On the other hand, specific pathophysiological processes may differ between species and subsequently exclude most of the animals if the study is aimed at developing new therapeutic options for medical application. The analysis of the data based on research studies shows that from 1950 to 1990, researchers looked for the optimal species that allows basic science and translational studies. During this time, authors reported data of animals from up to 27 different species (Fig. 3). Thereafter, however, physiological research switched completely, and many species were no longer used. In 2020, data were presented from only nine different species.

**Fig. 4:**
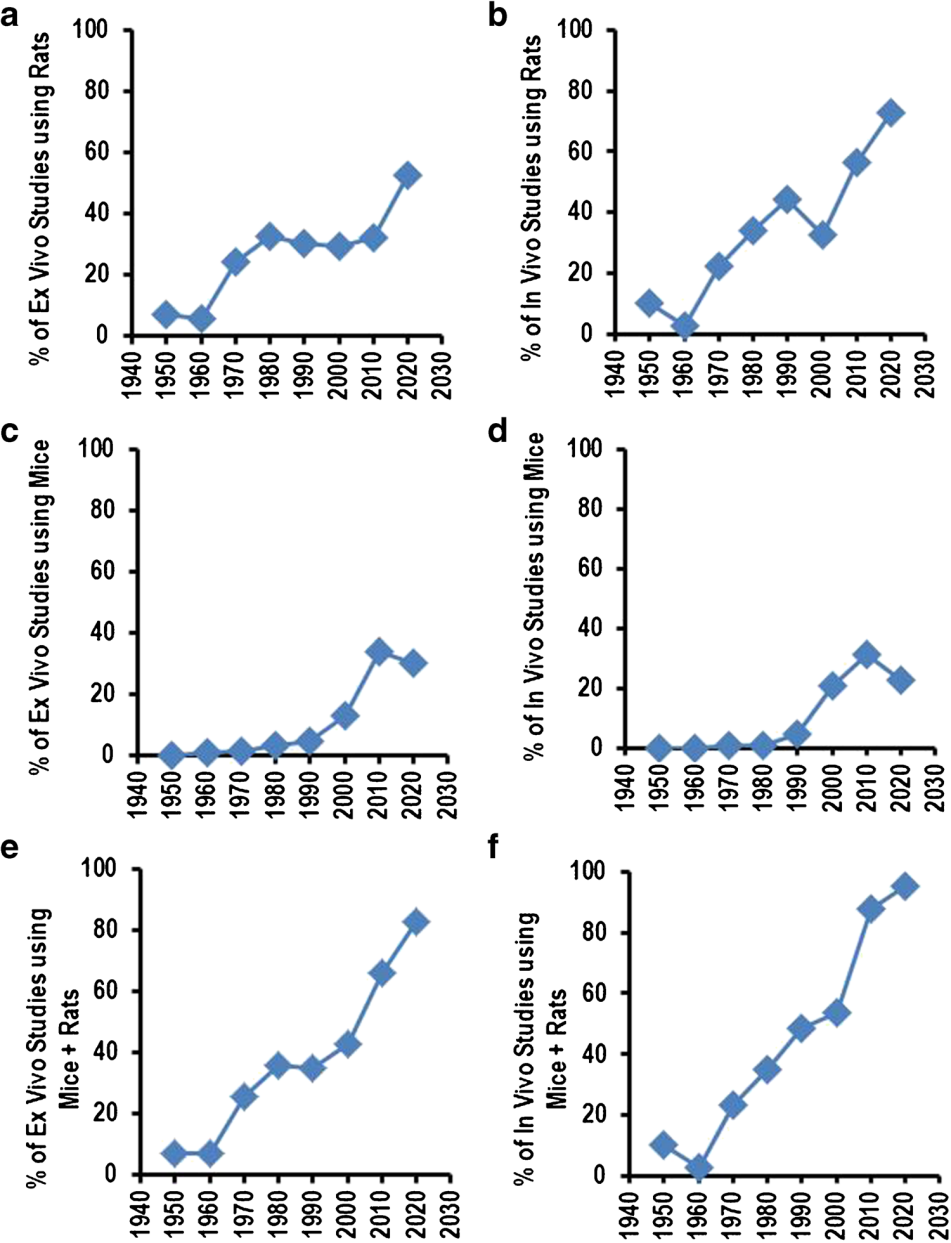
Use of small rodents in research publications published in *Pflügers Archiv – European Journal of Physiology* per year. **A**, **B** Data for rats. **C**, **D** Data for mice. **E**, **F** Data for rats and mice. Data expressed as % of all animal-based studies

At the same time, there began a focus on rodents, specifically rats and mice. From 1950 to 2020, the percentage of ex vivo studies using rats increased from 7.1 to 52.4% (Fig. 4A), and the percentage of in vivo studies using rats increased from 10.0 to 72.7%. In addition, 36.6% of all in vitro studies used rat-based material in 2020 (Fig. 4B). Mice were increasingly used from 1970 on, but they were used in a relevant number of ex vivo and in vivo studies not before 2000 (Fig. 4C, D). In our days, mice and rats account for 82.6% of all ex vivo studies and 95.4% of all in vivo studies in physiology (Fig. 4E, F). At the same time, the use of frogs, dogs, cats, and pigs that dominated physiological research before were largely replaced more or less completely by small rodents.

### Ethical approval

**Fig. 5:**
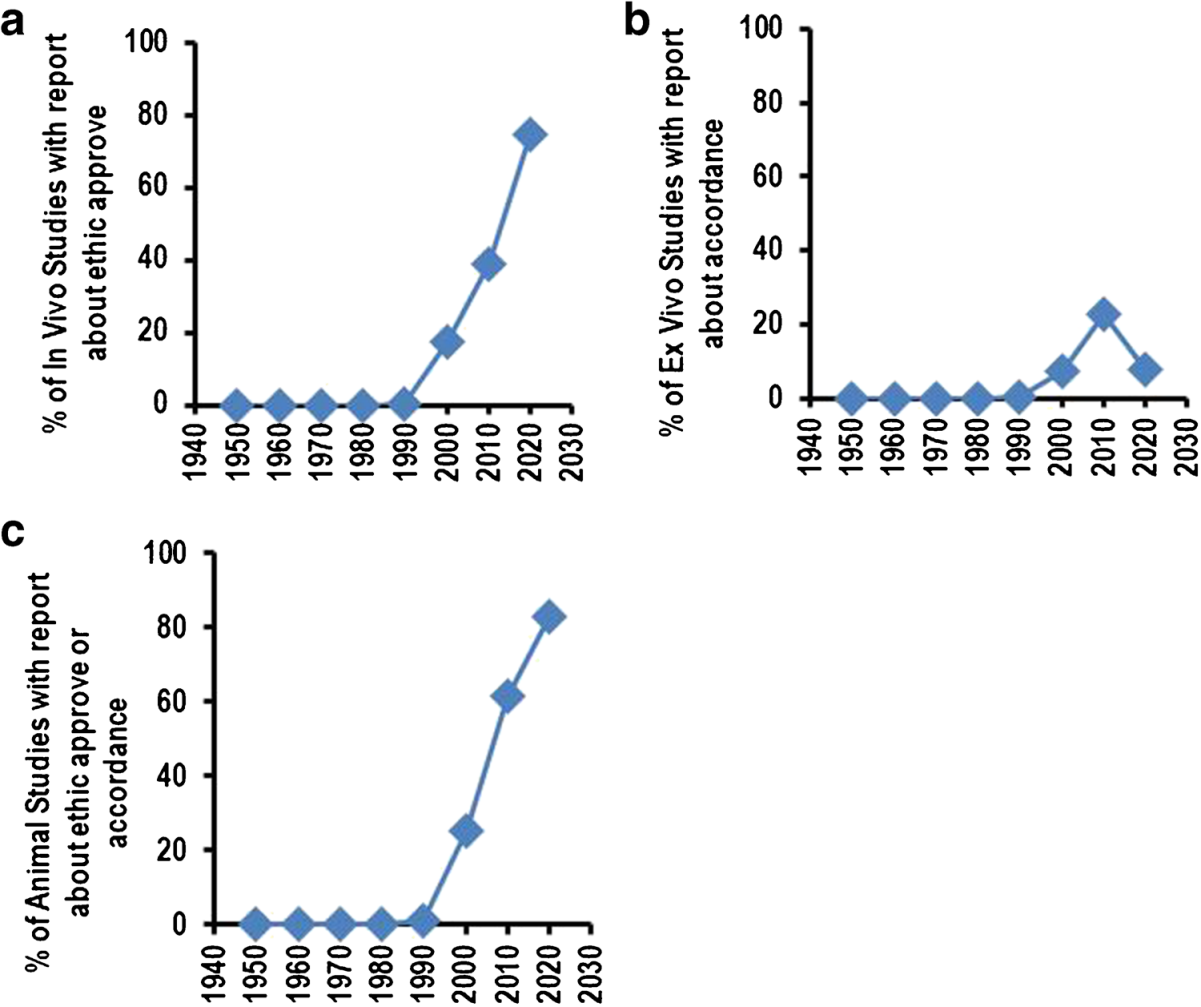
Inclusion about ethic reports. Data are expressed as % of all animal-based studies. **A** Data for % of in vivo studies reporting about ethic approve. **B** Data for % ex vivo studies reporting that they are in accordance with rule for animal handling. **C** Data for % of animal studies reporting that they are in accordance with general rules

In the last century, reports about ethical approval of individual studies were not required, or such information was not requested from the journal. Today, it is mandatory to get permission prior to the start of animal experiments, and it is also normally reported in the publication that such studies were approved. In 2020, 74.6% of all studies in the journal reported about permission (Fig. 5A). However, ex vivo studies also require animal housing. Animal housing and handling should be in accordance with the general rules of animal housing and methods used to sacrifice animals. As ex vivo studies are a relevant part of physiological science, part of all studies reported that their studies are in accordance with such assumptions (Fig. 5B). In total, 82.5% reported in 2020 about permission and/or accordance of their studies (Fig. 5C). The remaining rest are in vitro studies.

### Number of animals per study

**Fig. 6:**
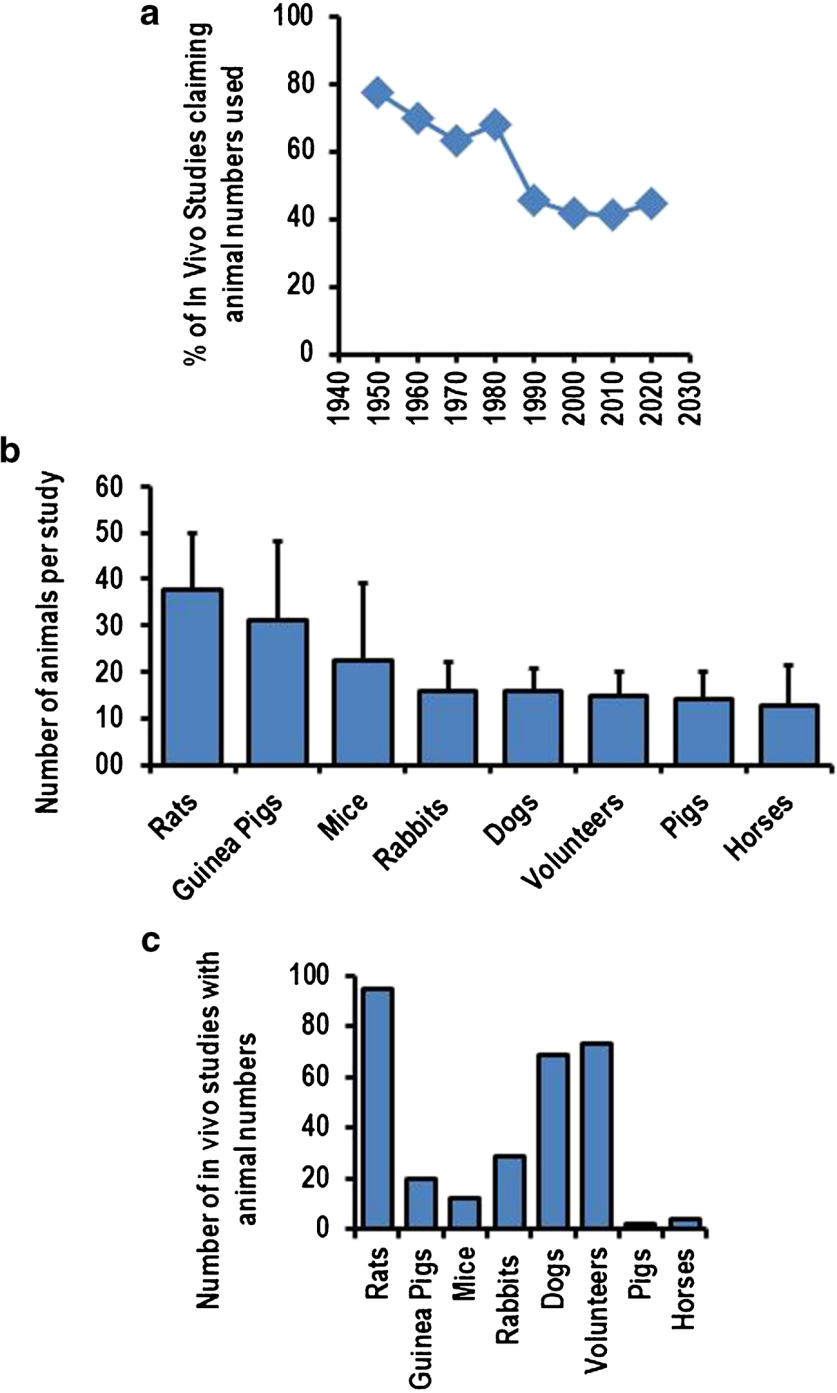
Reporting about animal numbers used per study. **A** % of animal-based in vivo studies claiming exact animal numbers. **B** Number of animals used in studies per species. **C** Total number of studies with animal species as indicated. Data in **B** are means ± S.D. of multiple studies using these species

**Fig. 7:**
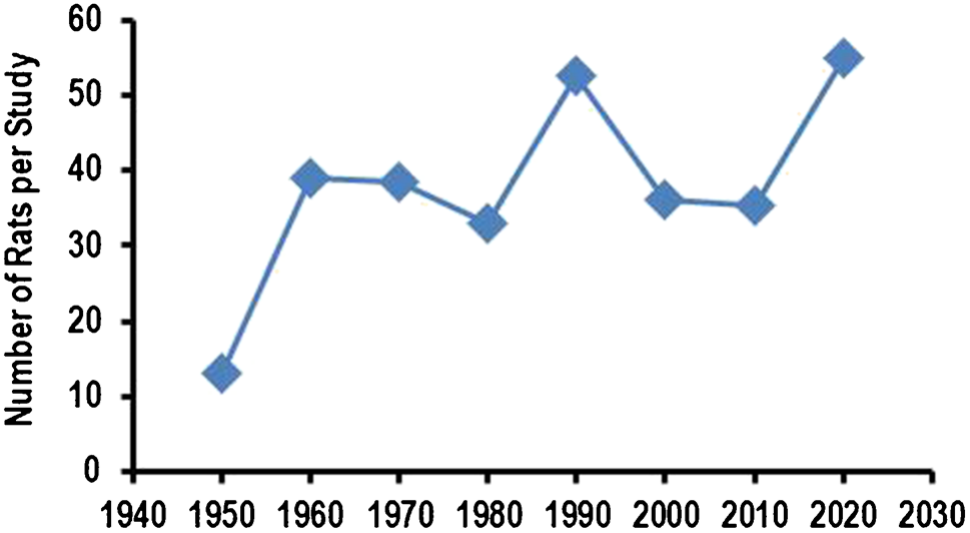
Number of rats per study in those research papers that give exact animal numbers

A central question in animal studies is whether robust results can be achieved with a lower number of animals used per study. This question is difficult to address from published studies. At first, the number of studies reporting about the absolute number of animals is often given only for in vivo studies, and even in these cases, only less than 50% of all published studies reported these numbers today (Fig. 6A). The percentage of studies reporting these data is declining during the last 60 years. Based on an analysis of these few studies and focusing on those species that are still used since 2000, the data show a clear tendency that fewer animals are used if the species are more developed. On average, researchers used more than 20 animals per study when reporting in vivo studies from rats, guinea pigs, or mice (Fig. 6B) but less than 20 animals when reporting about studies with rabbits, dogs, pigs, or horses. A similar number as for more developed animals was required for patient-based studies. Such an analysis is difficult because more than one variable affects these data: The lower number of studies reporting these data in the present compared to the past (Fig. 6A) and the higher number of studies dealing with rodents that more often do not report about exact numbers (Fig. 6C). This produces a bias in such type of analysis. However, as shown for rats as the most suitable animal model in physiology today, there is no trend that robust data leading to a full-paper publication can be achieved with a lower number of animals (Fig. 7).

### Comparison to other studies published in the journal of physiology

Finally, it was investigated whether the results obtained by an analysis of studies published in the *Pflügers Archiv – European Journal of Physiology* are for physiology in a more general view. Therefore, the studies published in the *Journal of Physiology* in volumes of 1960, 1980, 2000, and 2020 were analyzed in a similar way and compared to the data from the *Pflügers Archiv – European Journal of Physiology*.

**Fig. 8:**
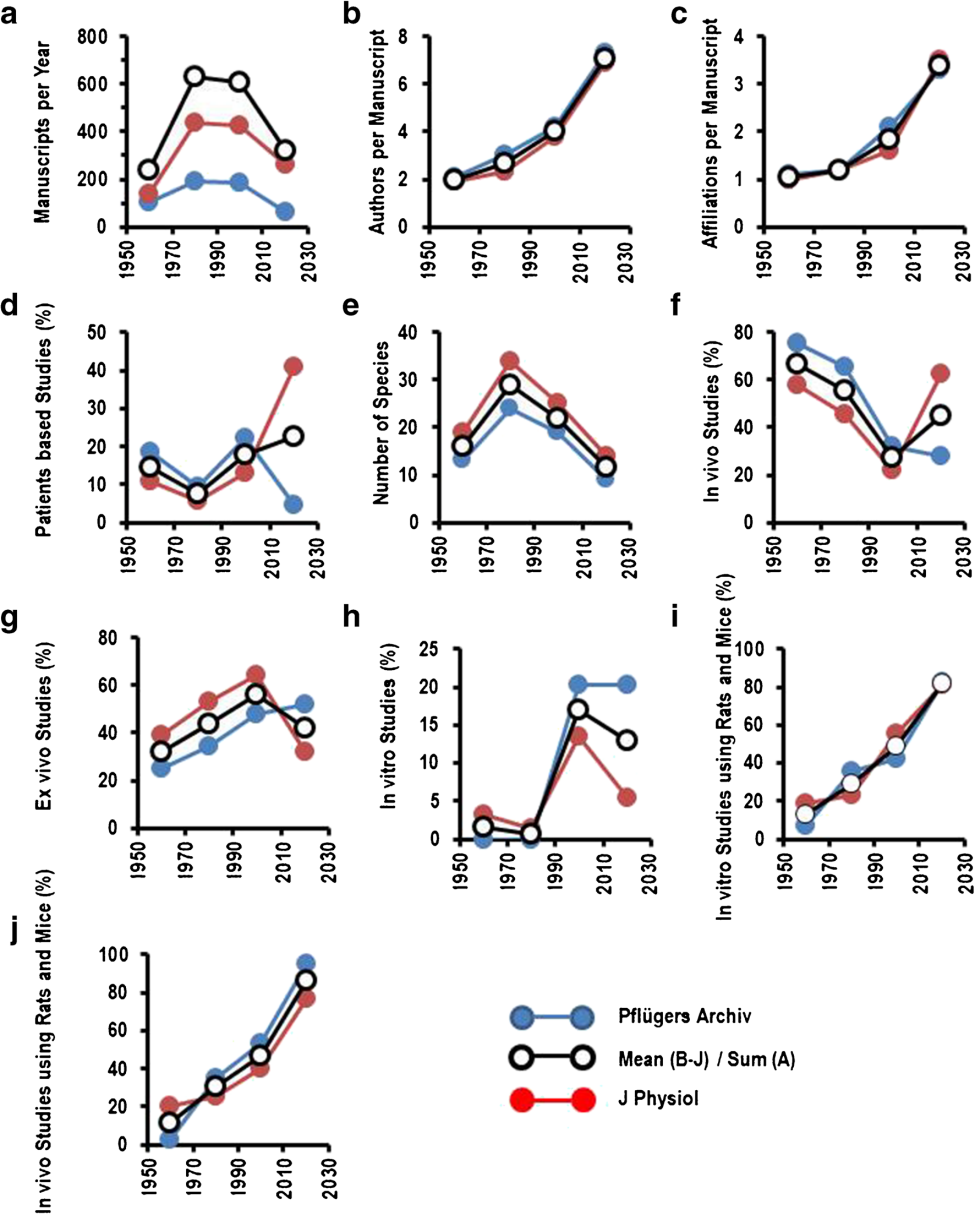
Comparison between *Pflügers Archiv – European Journal of Physiology* (*Pflügers Archiv*) and *Journal of Physiology* (*J Physiol*). Data are given separately for the two journals and as a mean (or sum in (**A**)) of the two journals that are compared. **A** Number of Manuscripts published per year. **B** Number of authors per manuscript. **C** Number of affiliations per manuscript. **D** % of patient-based studies. **E** Number of species used for studies. **F** % of in vivo studies. **G** % of ex vivo studies. **H** % of in vitro studies. **I** % of studies using mice and rats

Both journals published a smaller number of studies from 2000 on (Fig. 8A). In both journals, the number of authors per study (Fig. 8B) and the number of affiliations (Fig. 8C) per study increased in a nearly identical way. Therefore, publication statistics develops nearly identical in both journals. However, from 2000 on, the *Journal of Physiology* increased substantially the number of patient-based studies (Fig. 8D), whereas in the *Pflügers Archiv – European Journal of Physiology*, no such increase was observed. Concerning animal-based studies, both journals reported about data from multiple species until 1980, but this number declined thereafter (Fig. 8E). The *Journal of Physiology* published more in vivo-based studies and less ex vivo and in vitro studies from 2000 on (Fig. 8F-H). Among the animal-based studies that were performed in vitro or in vivo during the seven centuries investigated here, the percentage of studies using rats or mice increased in a nearly linear way, leading to numbers of more than 80% of animal-based studies that deal with rat or mouse at present (Fig. 8I, J).

## Discussion

This analysis investigated how restrictions in animal testing initially introduced by the publication of the 3R criteria affected physiological research. The study is based on published manuscripts that passed a peer-review process. That means experts in the field accepted these studies as scientifically sound and novel. The central question is how research is influenced by 3R, 3R + 3R, or 3R + 2R criteria. The main important finding of the study is that physiological science achieved major aims of the 3R strategy such as reduction, replacement, and refinement in the last 50 years. The relative contribution of in vivo studies decreased with time (reduction), and in vivo studies were replaced by in vitro and ex vivo studies. In addition, studies were transferred from dogs and cats to small rodents. However, it is also clear that at a certain level, scientific research in physiology cannot completely replace animal testing. Therefore, a stable proportion of 20% of all physiological studies that are published in the *Pflügers Archiv – European Journal of Physiology* is still using animal testing. Furthermore, the replacement of in vivo studies by ex vivo or in vitro does not necessarily reduce the number of animals required. Another important finding of this study is that the requirement to receive robust data does not reduce the number of animals per study. Finally, the data show also that physiological research in our days requires the cooperation of scientists with different expertise. This is the first scientific analysis about changes in animal use in physiology and therefore quite important for this discipline.

### Effect on reduction of animal testing

When we look at the studies published in the *Pflügers Archiv – European Journal of Physiology*, the percentage of in vivo animal studies in physiological science declined from 1970 to 1990. Before 1990, in vivo experiments were the dominant form of physiological research. However, starting from 1970 on, ex vivo studies replaced more and more in vivo experiments. In 2020, they counted for 50.9% of all studies in comparison to 27.8% in vivo studies. More importantly, this ratio is more or less stable during the last 40 years. Thus, it appears that approximately 20% of physiological research cannot be replaced by alternative methods. This conclusion is in line with suggestions given for Alzheimer’s and Parkinson’s disease research as well, and the current study extends this conclusion to a brought field of physiology [1]. Starting in 1990, a number of in vitro studies (now counting for 20.3% of all studies in 2020) replaced even ex vivo studies that further reduced the number of animals used in physiological science. Is this a consequence of the 3R strategy initiated in 1959? It is difficult to address this question properly, but we must keep in mind that patient-based studies also declined since 1950. It may be that the focus of physiologic research is directed into a more detailed analysis of organs and cells that drive this process rather than the acceptance of the 3R criteria that triggered this development. The 3R criteria were introduced approximately 20 years before a trend to reduction, replacement, and refinement was observed in physiology. It seems that 3R itself was not the main driver for this development, but science itself produced this effect. Which of these results are representative of physiology in a more general term? To address this point, data were compared to studies published in the *Journal of Physiology*. From 1960 to 2000, the development of animal use in physiological science was comparable when analyzing studies from both journals. There was a strong decline of in vivo studies and an upcoming publication of in vitro studies. During the last 20 years, the publications between both journals differed a little bit. In any way, it seems that in vivo studies are still required for clarifying open questions in approximately 20–30% of all studies, whereas in vitro studies contribute to a maximal of 20% of all studies from 1980 on.

### Effect on replacing in animal testing

As outlined above, the reduction of animal experiments in physiology is triggered by replacing in vivo experiments with ex vivo and in vitro studies. However, ex vivo studies still require the use of animals. Unfortunately, it is not common practice to report the number of animals used for such studies. This is a major problem both from the view of 3R and from the scientific view. If we do not know how many animals (thereby preparations) were used for a certain study, we do not know whether the replacement of in vivo experiments by ex vivo experiments reduces the number of laboratory animals used in physiological science. The scientific problem generated by this lack of report is that we have no idea how robust preparations from animals really are. That is, researchers who report that hearts for the Langendorff preparations (ex vivo analysis) were used report that they used organs only in those cases in which the initial left ventricular pressure reached a certain physiological value. However, they do not report at the same time how often they stopped their experiments. Another question is how representative are the published results when not all preparations can be used. Finally, the replacement of in vivo and ex vivo studies by in vitro studies (often cell cultures) ignores the fact that although no animal must be sacrificed to receive the cell material, nearly all such studies use serum from calves. Thus, these studies are not free from animal use. Scientifically, the different compositions of serum make it more difficult to repeat such experiments in another laboratory. In conclusion, this analysis shows a robust decline of in vivo studies but not necessarily a reduction in animal use. These limitations may explain the lack of further increase of in vitro studies published in physiological journals.

### Effect of refinement on animal testing

This aspect of the classical 3R is the one that shows the greatest difference in comparison to the past. As outlined above, in vivo experiments were largely replaced by ex vivo and in part by in vitro experiments. As a consequence, harm to animals is strongly reduced. In addition, another aspect of refinement is that animals with lower degree of development are used. After a period from 1950 to 1990 with the use of up to 25 different species, there is a clear reduction in species numbers used in physiology in our days. In fact, small rodents count now for more than 95% of all in vivo experiments and have replaced dogs, cats, frogs, sheep, or goats to claim some of them. Both journals were founded in the nineteenth century to publish studies in the physiology of people and animals. In our dates, both journals are transferred into journals for the physiology of small rodents. Thus, researchers, editors, and reviewers accept the advantages of small rodents. These are easy genetic modification, relatively low costs in handling, high reproducibility of animals, and easy handling of these animals. In combination with large similarities to human physiology, these advantages make small rodents an ideal model for many aspects of physiology.

### Data generation in physiology

The replacement of in vivo studies by ex vivo and in vitro studies goes along with a specialization of scientists. Whereas in the early post-war period normally one or two authors reported about data generated in one institute, we have now in general groups of scientists that generate data for a given study. They come from multiple affiliations. The current analysis quantified these data and surprisingly, although the number of studies is reduced in comparison to the last century more scientists report data in both journals today. This indicates the complexity of current research and also leads directly to a situation in which individuals cannot be easily identified with certain projects. On the other hand, specialization is required to generate robust data of high quality. How does physiological research react to the future challenges such as the requirement of robustness, registration, reporting, reproducibility, and rigor of the data? This study showed that the robustness of data generation requires a certain degree of data point per analysis. Therefore, we cannot expect a further reduction in animal numbers per study. Interestingly, the trend of the current publication suggests that a lower number of animals can be used in studies with larger animals. In other words, reduction will increase by the use of big animals but the use of more developed animals leads to ethical and also economic problems. Certainly, this is a dilemma in biomedical science. Registration of studies is required in our days. The question is, however, whether this will be reported in the publications. Registration, i.e., the requirement of ethical votes prior to animal testing, also leads to the participation of different local registration rules that directly affect the reproducibility of data from animal testing. This is another problem that has to be solved in the future. Reporting of data is also required. However, studies that generated new data will always have a higher chance of being published in well-recognized journals, and this generates a bias and lack of visibility of the reproducibility of data. Here, we will need alternative ways for publications because the current strategies of scientific journals do not support this way of publication. Reproducibility of data is considered a main problem, but to address this question properly, we need research that allows us to identify the points that are responsible for variability. Such studies are currently difficult to get published. We need research activities in the future that allow us to identify the causes that limit reproducibility. Finally, the rigor of data requires a better data presentation, i.e., original data must be given [11]. Here, we need an electronic add-on to publications that deliver these data. The current way that they have to be requested by the corresponding author is not really practicable as many scientists change their affiliations during their careers. Data handling is another problem as well.

### Limitations of this study

As mentioned before, the use of animal handling in physiological science is a dynamic process underlying multiple facets. This study describes the use of animals and animal-based sources in physiological science. New concepts such as ARRIVE 2.0 are aimed at improving the reproducibility of results generated by animal testing a major problem today. As they were published in 2020, they can hardly affect the results described here. The first version of ARRIVE was published 10 years before, but again, it is nearly impossible to decide the effect of this initiative on the results described here. Mainly, ARRIVE is aimed at improving the rigor and reproducibility of data achieved by animal-based experiments. However, the finding that the use of animals in physiological science during the last 70 years started to change 20 years after the publication of the 3R criteria may give us a hint that not the publication of the criteria forced these changes but the scientific development. Similarly, the acknowledgment of ethical statements for animal experiments in studies today indicates the sensitization of editors to this aspect of physiological studies, but not necessarily that similar considerations were performed in the last century as well. Even in times in which ethical approval was not required, it is clear that scientists already worked to similar criteria, thus choosing the best appropriate animal model. This is clear by the high number of different species used for physiological science during the last century that is now replaced by a nearly exclusive use of small rodents.

Another obvious limitation of this study is that it is difficult to decide how innovation and new technologies affect the outcome of this analysis. That is, how can we know whether the genetic modification of mice triggered new technologies to analyze functional data in a small animal such as a mouse or whether new technologies triggered the use of mice independent of the possibility of genetic modification? In principle, genetic manipulation can be performed in all types of animals, but the cost efficiency of using mice may have forced the subsequent technical improvements to use these small rodents. In any way, a clear preference to use mice instead of rats cannot be seen in physiological studies.

It is also important to note that from published data, we cannot calculate the total number of animals used in research. As mentioned before, often the total number of animals used for the study is not given, and even more importantly, they are normally not given for ex vivo studies. Moreover, as it is more likely that studies are published that confirm a given hypothesis than those that reject the hypothesis, there is a large number of unreported cases. This study can only investigate how animals were used in studies that were finally accepted as scientific sound and important.

## Conclusion

Based on the 3R strategy, as they were announced by researchers and by science itself, the use of animals in physiological science has dramatically changed in the last 70 years. Replacement, reduction, and refinement were achieved to a certain level. However, it seems that animal testing cannot totally be replaced and is still required for biomedical science and physiology in particular. Future strategies must consider better that improvement in animal handling does not lead to a reduction in data robustness. Furthermore, research is required to improve the reproducibility of experiments and generate rigor of data as a prerequisite to improve our physiological view of life and develop new therapeutic options for the medical challenges of the future.

## Data Availability

No datasets were generated or analysed during the current study.
